# Cyclin-dependent kinase-inhibitor screening and structural analysis identify a CDK-like kinase candidate in *Naegleria fowleri*

**DOI:** 10.1186/s13071-026-07525-8

**Published:** 2026-06-24

**Authors:** Min-Jeong Kim, Joo Hwan No

**Affiliations:** 1 Department of Parasitology, School of Medicine, Chungbuk National University, Cheongju, Republic of Korea; 2 Chemical and Structural Biology of Pathogens Laboratory, Discovery Biology, Institut Pasteur Korea, Seongnam-Si, Gyeonggi-Do 13488 Republic of Korea; 3 Department of Advanced Drug Discovery and Development, Institut Pasteur Korea School, University of Science and Technology (UST), 16 Daewangangyo-Ro 712 Beon-Gil, Bundang-Gu, Seongnam-Si, Gyeonggi-Do 13488 Republic of Korea

**Keywords:** Primary amebic meningoencephalitis, *Naegleria fowleri*, Cyclin-dependent kinase, Drug repurposing, Molecular docking, Molecular dynamics

## Abstract

**Background:**

Primary amebic meningoencephalitis (PAM) caused by *Naegleria fowleri* is a highly fatal central nervous system infection for which effective treatment options remain limited. This study explored CDK-related pathways in *N. fowleri* using a focused CDK inhibitor library and structure-based analysis of a selected CDK-like protein.

**Methods:**

A library of 126 CDK inhibitors was screened against *N. fowleri* trophozoites using a luminescence-based ATP viability assay, and active compounds were further evaluated in dose–response assays. Human CDK1–9 sequences were used for BLASTp searches against the *N. fowleri* proteome, followed by comparative sequence analysis with *Trypanosoma brucei* cdc2-related kinases (CRKs). Nf_CDK-like protein1 (FDP41_013684) was selected for homology modeling, molecular docking, and 100-ns molecular dynamics simulations.

**Results:**

Screening identified 23 active CDK inhibitors with IC_50_ values ≤ 10 μM against *N. fowleri*, including five prioritized compounds with submicromolar activity: AZD5438, GSK-3 inhibitor IX, milciclib, roniciclib, and SU9516 (IC_50_, 0.30–0.88 μM). Sequence analysis identified five CDK-like proteins in the *N. fowleri* proteome, among which Nf_CDK-like protein1 showed the highest similarity to *T. brucei* CRK3 and shared approximately 58–61% sequence identity with human CDK1/2. Conserved kinase features, including the glycine-rich loop, αC-helix, hinge region, DFG motif, and activation loop, were retained in Nf_CDK-like protein1. Docking analysis placed all five prioritized compounds within the predicted ATP-binding cleft, with shared interactions around Lys60, Tyr42, Asp113, and the hinge-proximal residues Ile110 and Asp111. Molecular dynamics simulations showed generally stable protein backbones and ligand poses over 100 ns.

**Conclusions:**

This study identified multiple CDK inhibitor scaffolds with potent in vitro activity against *N. fowleri* and supports Nf_CDK-like protein1 as a plausible CDK-like kinase target candidate through integrated phenotypic and structure-based analyses, with direct biochemical validation needed in future studies. These findings suggest that CDK-like pathways may represent a relevant molecular axis in *N. fowleri* and provide a basis for future structure-guided optimization of anti-amoebic candidates.

**Graphical Abstract:**

**Figure:**
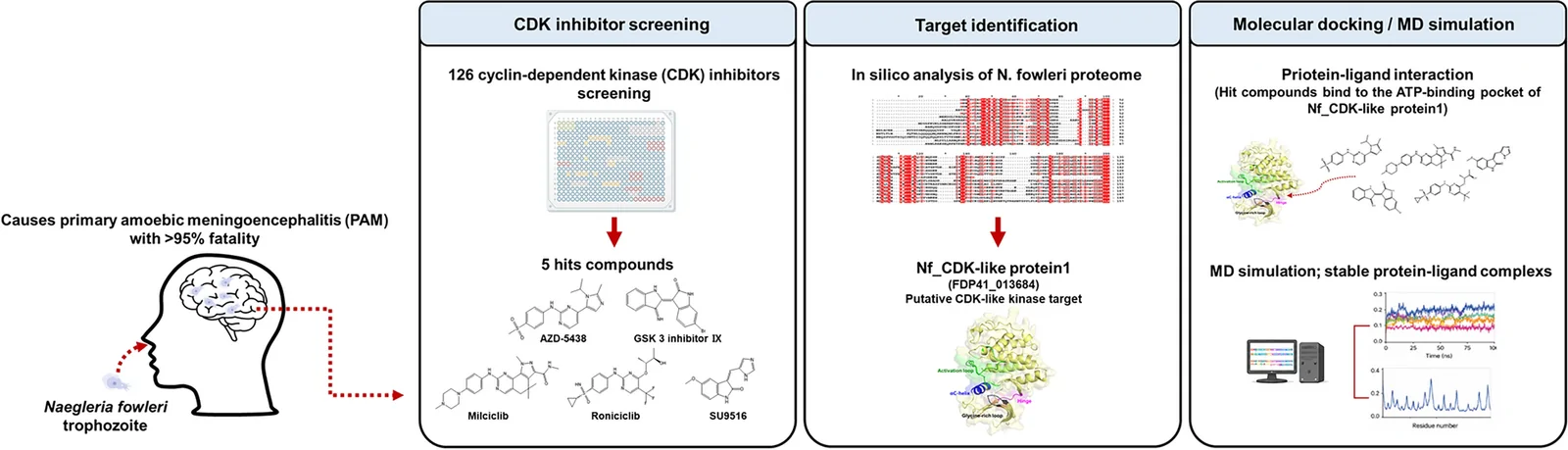

**Supplementary Information:**

The online version contains supplementary material available at https://doi.org/10.1186/s13071-026-07525-8.

## Background

*Naegleria fowleri* is a thermophilic free-living amoeba distributed in warm freshwater, soil, and geothermal environments. Infection occurs when contaminated water enters the nasal cavity, allowing the amoeba to migrate along the olfactory route into the central nervous system and cause primary amoebic meningoencephalitis (PAM) [1–3]. Although PAM is a rare disease, it progresses rapidly and is associated with a fatality rate exceeding 95% [1–3]. Current clinical treatment generally relies on multidrug regimens that include Amphotericin B and miltefosine in combination with additional agents, such as azoles, macrolides, and rifampin [2, 4–7]. However, the available evidence is largely limited to case reports and small case series, and no standardized therapy with reproducible efficacy has yet been established [4–7]. In addition, amphotericin B is associated with substantial toxicity, and maintaining adequate drug exposure in central nervous system infection remains challenging [1, 2, 7]. Therefore, the development of new therapeutics against *N. fowleri* requires the identification of therapeutically relevant molecular targets.

Several phenotypic screening and drug repurposing studies have identified compounds with in vitro activity against *N. fowleri*, including F1FO adenosine triphosphate synthase inhibitor leads, indolocarbazole derivatives, and miconazole-like scaffolds developed toward *N. fowleri* sterol 14α-demethylase (NfCYP51) [8–11]. In addition, target-based studies have suggested exploitable metabolic vulnerabilities, particularly the sterol biosynthesis pathway and the mevalonate/prenylation pathway, including 3-hydroxy-3-methylglutaryl-coenzyme A reductase and protein farnesyltransferase [10–12]. However, studies on target proteins that could inhibit *N. fowleri* remain limited, and proteins associated with the proliferation of *N. fowleri* have not yet been sufficiently investigated [8–12].

Cyclin-dependent kinases (CDKs) are a major family of serine/threonine protein kinases that regulate essential eukaryotic processes, including cell-cycle progression and transcription [13–15]. In humans, CDK1, CDK2, CDK4, and CDK6 mainly regulate cell-cycle progression, whereas CDK7, CDK8, CDK9, CDK12, and CDK13 are involved in transcriptional regulation [13–16]. Human CDKs have been extensively studied as therapeutic targets, and numerous small-molecule CDK inhibitors have been developed [17–20]. In protozoan parasites, CDK- or CRK-related kinases have been implicated in parasite growth, division, and survival, and have, therefore, been considered pharmacologically relevant targets [21–25]. In trypanosomatids, proliferation-associated CRKs are functionally important and have been studied as pharmacological targets, and CRK-directed inhibitors have been reported to show both kinase-inhibitory activity and antiparasitic effects [21–25]. However, proliferation-associated molecular targets remain insufficiently defined in *N. fowleri*. These findings support CDK-like kinases as a target class for investigating growth-associated pathways and for therapeutic development in *N. fowleri.*

We selected a focused CDK inhibitor library as a chemically and biologically relevant set for exploring proliferation-associated kinase pathways in *N. fowleri*. We screened 126 CDK inhibitors against *N. fowleri* trophozoites and combined phenotypic screening with sequence- and structure-based analyses to investigate the drug-target potential of previously uncharacterized CDK-like kinases. This study provides the first CDK-focused screening and structural analysis in *N. fowleri*. Homology searches identified several CDK-like proteins in the *N. fowleri* proteome, and these candidates were further compared with *Trypanosoma brucei* CRKs to assess conserved kinase-domain features. Among these candidates, Nf_CDK-like protein1 (FDP41_013684) was selected for detailed structural analysis. By characterizing the interactions between prioritized inhibitors and Nf_CDK-like protein1, we aimed to define structural features relevant to inhibitor binding and to provide a basis for the future optimization of CDK inhibitor-based anti-amoebic candidates.

## Methods

### Compounds and reagents

Amphotericin B and miltefosine were purchased from Sigma-Aldrich (St. Louis, MO, USA). A focused library of 126 cyclin-dependent kinase (CDK) inhibitors was obtained from MedChemExpress (Monmouth Junction, NJ, USA). All compounds were dissolved in dimethyl sulfoxide (DMSO; Sigma-Aldrich) to prepare stock solutions according to the manufacturers’ recommendations.

### Naegleria fowleri cultivation

*Naegleria fowleri* Carter NF69 strain (ATCC 30215) was obtained from the American Type Culture Collection (ATCC, Manassas, VA, USA). Trophozoites were maintained axenically in Nelson’s medium supplemented with 10% (v/v) heat-inactivated fetal bovine serum (FBS; Gibco, Thermo Fisher Scientific, Waltham, MA, USA) and 1% penicillin–streptomycin (Gibco) at 37 °C, as previously described [26, 27]. Cultures were monitored daily and passaged into fresh medium when they reached approximately 80% confluence.

### Anti-amoebic activity screening against *N. fowleri*

Anti-amoebic activity was quantified using a luminescence-based adenosine triphosphate (ATP) viability assay (CellTiter-Glo 2.0; Promega, Madison, WI, USA) [28]. *N. fowleri* trophozoites were seeded in 384-well plates at 3 × 10^3^ cells/well and incubated with test compounds for 48 h at 37 °C. In the primary screen, all compounds were tested at a single concentration of 10 µM. Compounds identified as primary hits were then evaluated in dose–response format using 10-point, twofold serial dilutions ranging from 25 µM to 24.4 nM. All assays were performed in duplicate, and the final concentration of DMSO was fixed at 0.5% (v/v). Wells containing 0.5% DMSO alone were used as negative controls, whereas Amphotericin B (50 µM) was included as a positive control. Luminescence was measured using a SpectraMax i3x microplate reader (Molecular Devices, San Jose, CA, USA). Percent inhibition and half-maximal inhibitory concentration (IC_50_) values were determined by nonlinear regression analysis in GraphPad Prism version 8.0 (GraphPad Software, San Diego, CA, USA).

### In vitro cytotoxicity assay

Cytotoxicity was evaluated using murine macrophage J774A.1 cells (ATCC TIB-67) [29]. Cells were seeded in 384-well plates at 5 × 10^3^ cells/well and allowed to stabilize overnight. Compounds were applied as serial dilutions and incubated for 48 h, after which cell viability was determined using CellTiter-Glo 2.0. Half-maximal cytotoxic concentration (CC_50_) values were calculated by nonlinear regression analysis in GraphPad Prism 8.0.

### Sequence analysis

Amino acid sequences of human CDK 1–9 were retrieved from the National Center for Biotechnology Information (NCBI) Protein database and used as queries for BLASTp searches against the *N. fowleri* protein set (Table 1). Candidate proteins were prioritized on the basis of overall sequence similarity and the presence of conserved kinase-domain motifs, and five proteins were designated Nf_CDK-like protein1–5. For comparative analysis of conserved cyclin-dependent kinase/cdc2-related kinase (CDK/CRK) features in protozoa, amino acid sequences of *Trypanosoma brucei* CRK1, CRK2, CRK3, and CRK12 were retrieved from the NCBI Protein and TriTrypDB [30]. These CRKs were selected as representative *T. brucei* CDK-related kinases previously associated with cell-cycle progression or parasite proliferation [21–25]. Conservation among human CDKs, Nf_CDK-like candidates, and *T. brucei* CRKs was examined by multiple sequence alignment using ClustalX version 1.8, with emphasis on residues associated with inhibitor recognition.

**Table 1:** Human CDKs, *T. brucei* CRKs, and *N. fowleri* CDK-like protein candidates used in the comparative sequence analysis

No	Description	Protein name	Locus ID	Size (aa)
1	Hs_CDK1	cyclin-dependent kinase 1 isoform 1_Homo sapiens	NP_001307847^a^	297
2	Hs_CDK2	cyclin-dependent kinase 2 isoform 1_Homo sapiens	NP_001789^a^	298
3	Hs_CDK3	cyclin-dependent kinase 3_Homo sapiens	NP_001249^a^	305
4	Hs_CDK4	cyclin-dependent kinase 4 isoform 1_Homo sapiens	NP_000066^a^	303
5	Hs_CDK5	cyclin-dependent kinase 5_Homo sapiens	AAL15435^a^	292
6	Hs_CDK6	cyclin-dependent kinase 6_Homo sapiens	NP_001138778^a^	326
7	Hs_CDK7	cyclin-dependent kinase 7_Homo sapiens	KAI4021525^a^	309
8	Hs_CDK8	cyclin-dependent kinase 8_Homo sapiens	KAI4062893^a^	464
9	Hs_CDK9	cyclin-dependent kinase 9_Homo sapiens	KAI4008569^a^	372
10	Tb_CRK1	cdc2-like protein kinase 1	CAA45595.1^a^	301
11	Tb_CRK2	cdc2-related protein kinase 2	CAA52676.1^a^	345
12	Tb_CRK3	cdc2-related kinase 3	CAA52688.1^a^	311
13	Tb_CRK12	cdc2-related kinase 12	Tb927.11.12310^b^	769
14	Nf_CDK-like protein1	Uncharacterized protein	FDP41_013684^a^	320
15	Nf_CDK-like protein2	Uncharacterized protein	FDP41_011785^a^	339
16	Nf_CDK-like protein3	Uncharacterized protein	FDP41_000261^a^	332
17	Nf_CDK-like protein4	Uncharacterized protein	FDP41_009938^a^	507
18	Nf_CDK-like protein5	Uncharacterized protein	FDP41_003847^a^	444

^a^ Human CDK sequences and *N. fowleri* CDK-like protein candidates were retrieved from the NCBI database. ^b^ *T. brucei* CRK sequences were retrieved from the TriTrypDB database.

*Hs* Homo sapiens, *Nf* *Naegleria fowleri*, *Tb* *Trypanosoma brucei*

### Homology modeling and ligand preparation

A three-dimensional (3D) homology model of the selected target protein was generated using the SWISS–MODEL server. Model stereochemistry was evaluated by Ramachandran plot analysis, and the overall fold and ATP-binding cleft architecture were inspected in PyMOL version 3.1.6.1 (Schrödinger, LLC, New York, NY, USA). Two-dimensional (2D) structures of the selected compounds were drawn in ChemDraw version 16.0 (PerkinElmer, Waltham, MA, USA), converted into 3D structures in Chem3D, and energy-minimized to relieve unfavorable steric contacts [31]. The minimized ligands were saved in Protein Data Bank (PDB) format. Ligands were prepared for docking in AutoDockTools (ADT) by adding polar hydrogens, assigning partial charges, and converting the files to Protein Data Bank, Partial Charge, and Atom Type (PDBQT) format.

### Molecular docking analysis

Molecular docking analyses were performed using AutoDock Vina version 1.2.7 (Center for Computational Structural Biology, Scripps Research, La Jolla, CA, USA), with file preparation and docking runs conducted through ADT [32]. Docking calculations were performed against the predicted ATP-binding cleft of the homology model. The docking grid was centered at x = − 25.054, y = − 23.284, and z = − 19.663 Å. For each ligand, multiple binding poses were generated, and the pose with the lowest predicted binding affinity was selected for further analysis. Docked conformations were visualized in PyMOL and BIOVIA Discovery Studio Visualizer, and protein–ligand contacts, including hydrogen bonds and hydrophobic interactions around the hinge region and adjacent pocket residues, were examined.

### Molecular dynamics simulations

Molecular dynamics (MD) simulations were performed for the apo protein and protein–ligand complexes to evaluate the stability of the docked binding poses and the dynamic behavior of the selected Nf_CDK-like protein. All simulations were carried out in GROMACS version 2026.0 using the CHARMM36m force field for the protein, and the systems were constructed with CHARMM–GUI Solution Builder [33, 34]. Each system was solvated in a periodic cubic box containing TIP3P water molecules, with a minimum solute-to-box distance of 10 Å. Systems were neutralized and adjusted to 0.15 M KCl by adding K + and Cl − ions [35]. After energy minimization using the steepest descent algorithm until the maximum force fell below 1000 kJ·mol − 1·nm − 1, equilibration was performed before production runs [36]. Production simulations were conducted for 100 ns with a 2 fs time step under isotropic pressure coupling using the C-rescale barostat. Long-range electrostatic interactions were treated using the particle mesh Ewald (PME) method, and bonds involving hydrogen atoms were constrained. Simulations were run under GPU acceleration on an NVIDIA GeForce RTX 4060, and trajectory coordinates were written every 50,000 steps (100 ps intervals). Protein structural stability was evaluated by backbone root mean square deviation (RMSD), ligand pose stability by ligand RMSD after least-squares fitting to the protein backbone, and residue-level flexibility by Cα root mean square fluctuation (RMSF). RMSF profiles of the five protein–ligand complexes were compared with that of the apo protein. Protein–ligand hydrogen bonds were analyzed using gmx hbond with standard geometric criteria, and both time-dependent hydrogen-bond counts and occupancies were extracted for comparison.

## Results

### Identification of active CDK inhibitors against *N. fowleri*

**Fig. 1:**
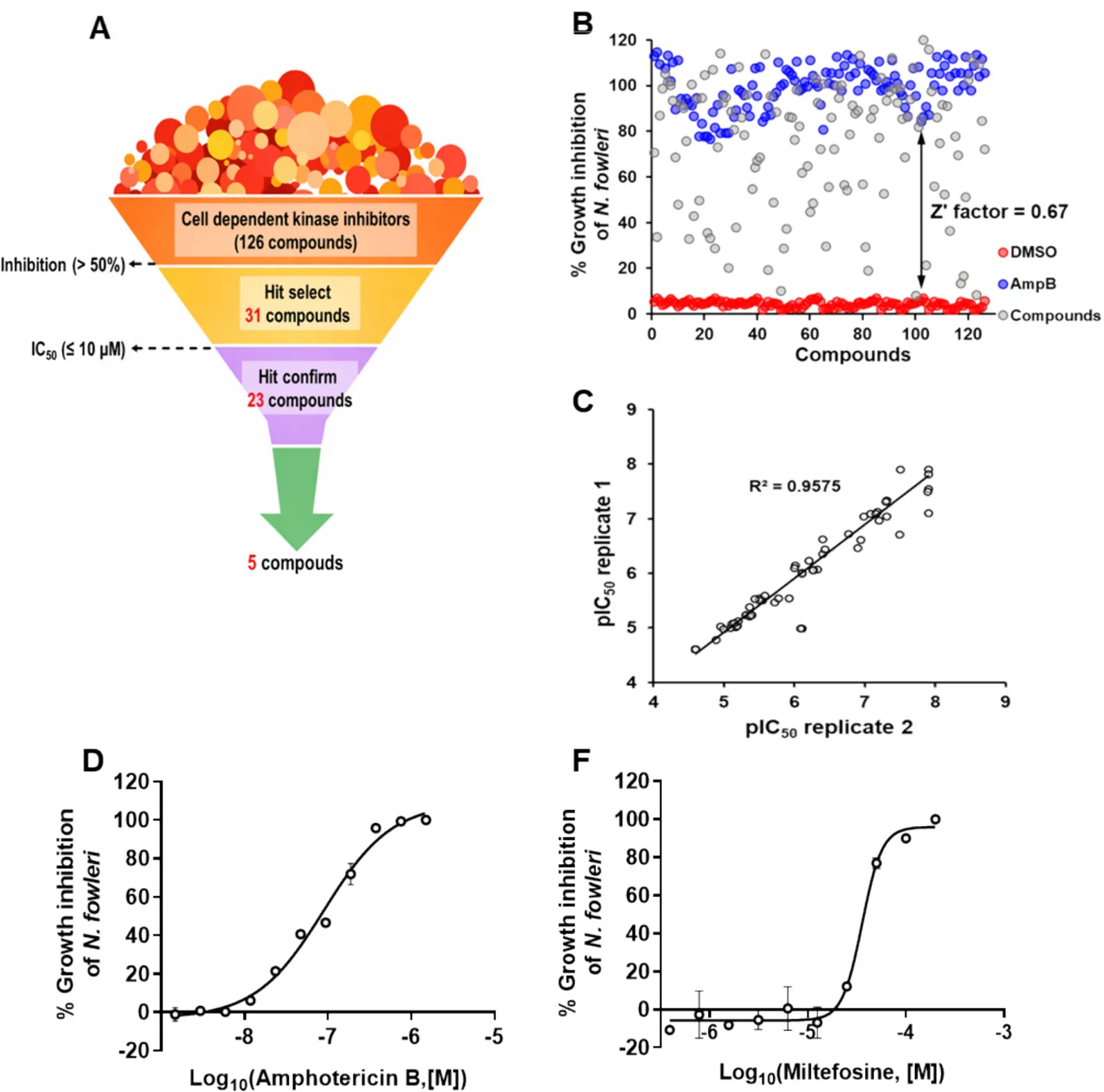
Phenotypic screening of the CDK inhibitor library against *N. fowleri*. **A** Schematic workflow of the 384-well ATP-based viability assay. **B** Primary screening results for 126 compounds tested at 10 μM. The dashed line indicates the 50% growth-inhibition threshold used to define primary hits; the Z′-factor was 0.67. **C** Correlation of IC_50_ values obtained from two independent experiments (R^2^ = 0.9778). **D** Dose–response curves of the reference drugs Amphotericin B and miltefosine

A focused library of 126 CDK inhibitors was screened against *N. fowleri* trophozoites using a 384-well, luminescence-based ATP viability assay (Fig. 1A). Compounds producing at least 50% growth inhibition at 10 µM were classified as primary hits. Assay performance was supported by a Z′-factor of 0.67 (Fig. 1B), and dose–response measurements showed high reproducibility between independent duplicate experiments (R^2^ = 0.9778; Fig. 1C). Amphotericin B and miltefosine, utilized as reference compounds, produced sigmoidal concentration–response curves, with IC_50_ values of 89 nM and 34.3 µM, respectively (Fig. 1D; Table 2).

**Table 2:** IC₅₀ values of the five prioritized hit compounds and reference drugs against *N. fowleri* trophozoites

No	Compounds	IC_50_ (µM) ± SD
1	Amphotericin B	0.089 ± 1.7253
2	Miltefosine	34.3 ± 2.545584
3	AZD5438	0.4645 ± 1.6491
4	GSK 3 inhibitor Ⅸ	0.7813 ± 0.2316
5	Milciclib	0.3027 ± 0.1234
6	Ronciclib	0.8828 ± 0.1437
7	SU9516	0.8340 ± 0.0750

Data are presented as mean ± SD from independent duplicate experiments

### Prioritization of five potent hit compounds

**Fig. 2:**
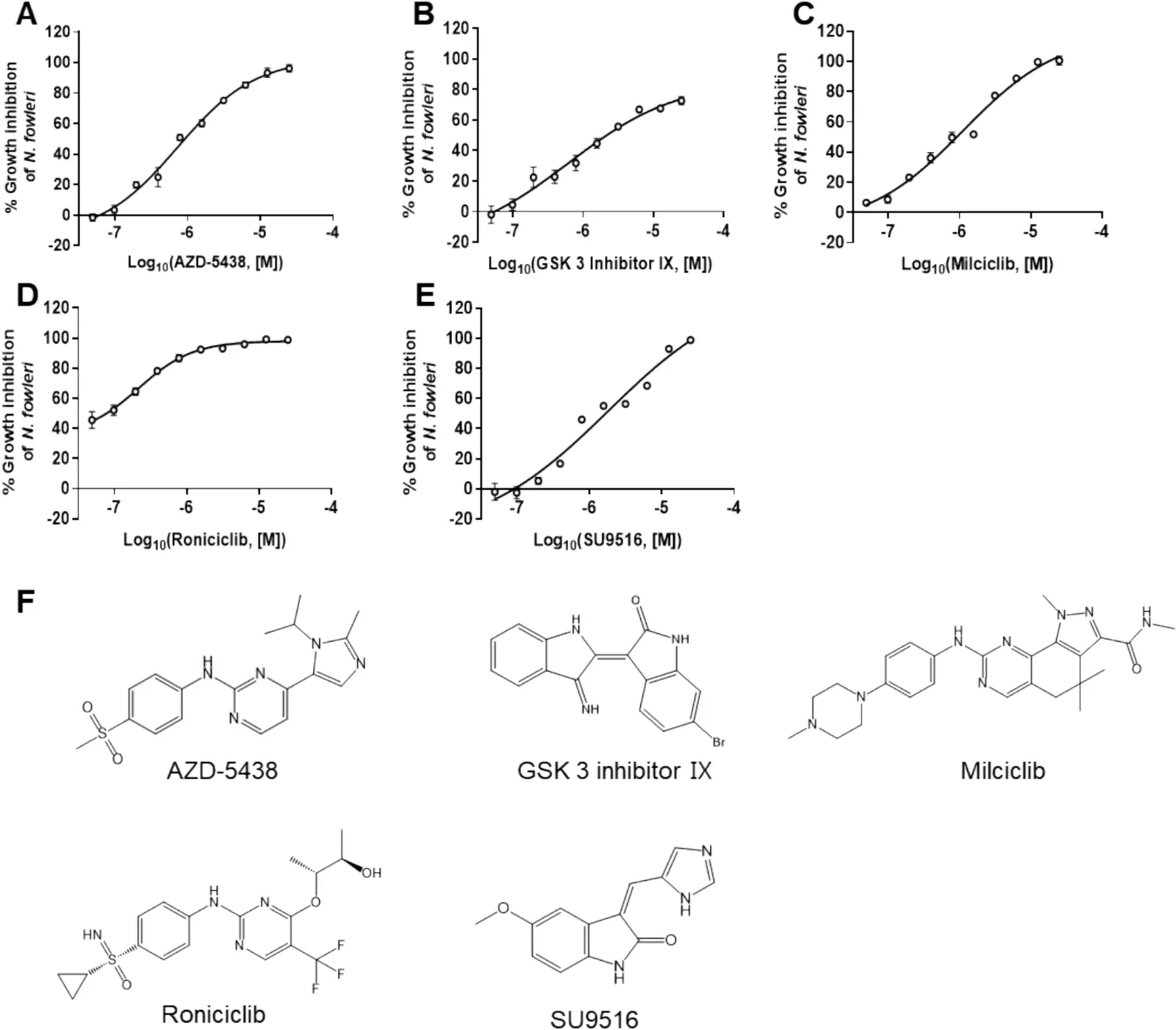
Dose–response curves and chemical structures of the five prioritized hit compounds. Viability curves of **A** AZD5438, **B** GSK-3 inhibitor IX, **C** Milciclib, **D** Roniciclib, and **E** SU9516 against *N. fowleri* trophozoites. **F** Chemical structures of the five prioritized compounds

Secondary dose–response testing of the primary hits identified 23 compounds with IC_50_ values ≤ 10 µM (Additional file 1: Table S1). Among these, AZD5438, GSK-3 inhibitor IX, milciclib, roniciclib, and SU9516 were selected for further analysis, because they reproducibly inhibited trophozoite viability in a concentration-dependent manner and displayed submicromolar potency, with IC_50_ values ranging from 0.3027 to 0.8828 µM (Fig. 2A–E; Table 2). Structural classification showed that AZD5438 and roniciclib belong to the anilino–pyrimidine class, whereas SU9516 and GSK-3 inhibitor IX contain indole-derived scaffolds; milciclib was grouped within the pyrimidine-containing kinase-inhibitor set (Fig. 2F). In J774A.1 cells, the five compounds showed CC_50_ values of 0.4–2.2 µM and selectivity indices (SI) of 1.03–2.85 (Additional file 1: Table S2 and Additional file 2: Figure S1).

### Selection of CDK-like protein of *N. fowleri* as a structure-based target candidate

**Fig. 3:**
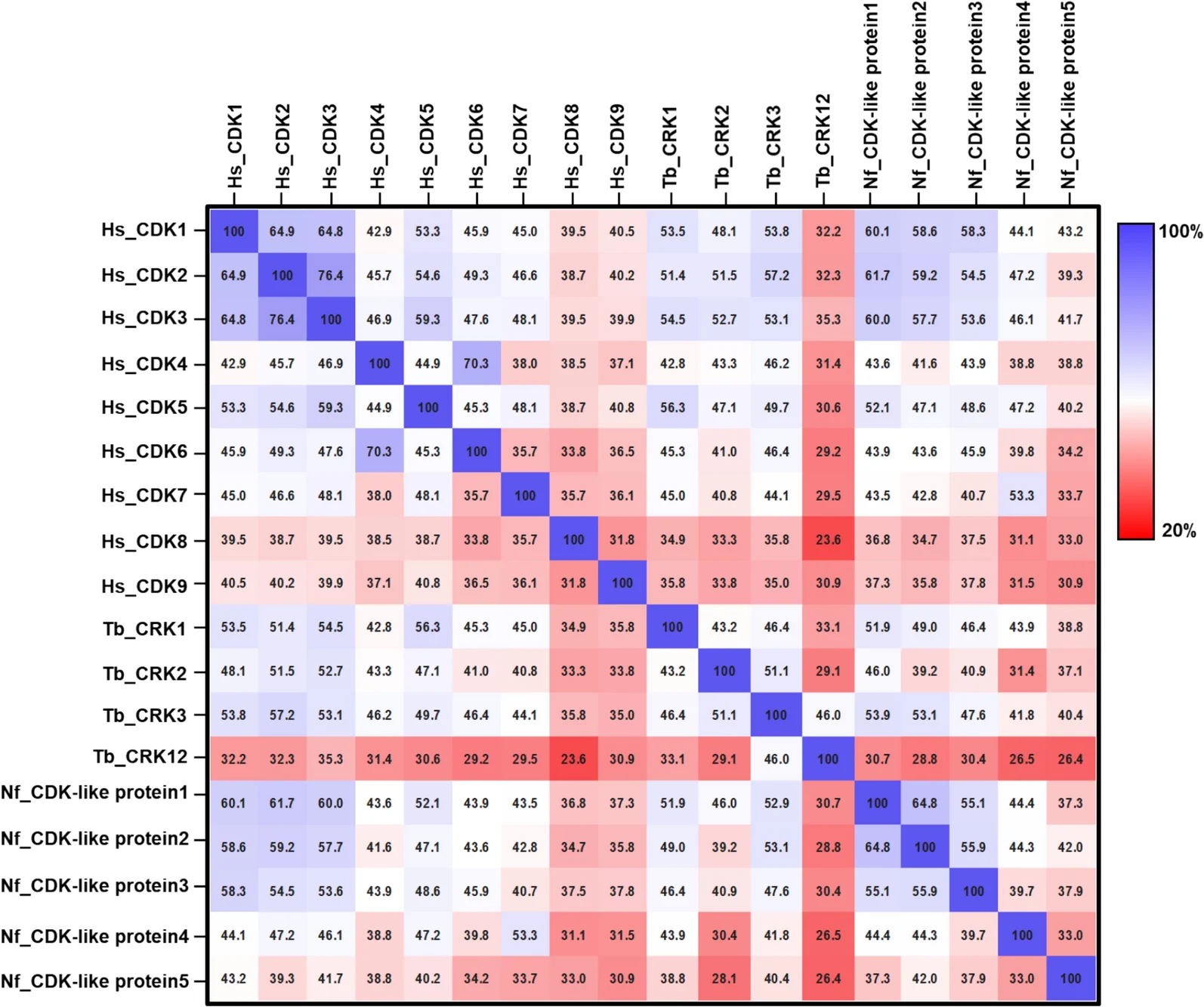
Sequence identity matrix of Nf_CDK-like protein candidates 1–5. Heatmap showing the percentage amino acid sequence identity among human CDKs (Hs_CDK1–9), *T. brucei* CRKs (Tb_CRK1, Tb_CRK2, Tb_CRK3, Tb_CRK4, and Tb_CRK6), and *N. fowleri* CDK-like protein candidates 1–5. Blue indicates higher sequence identity, and red indicates lower sequence identity

**Fig. 4:**
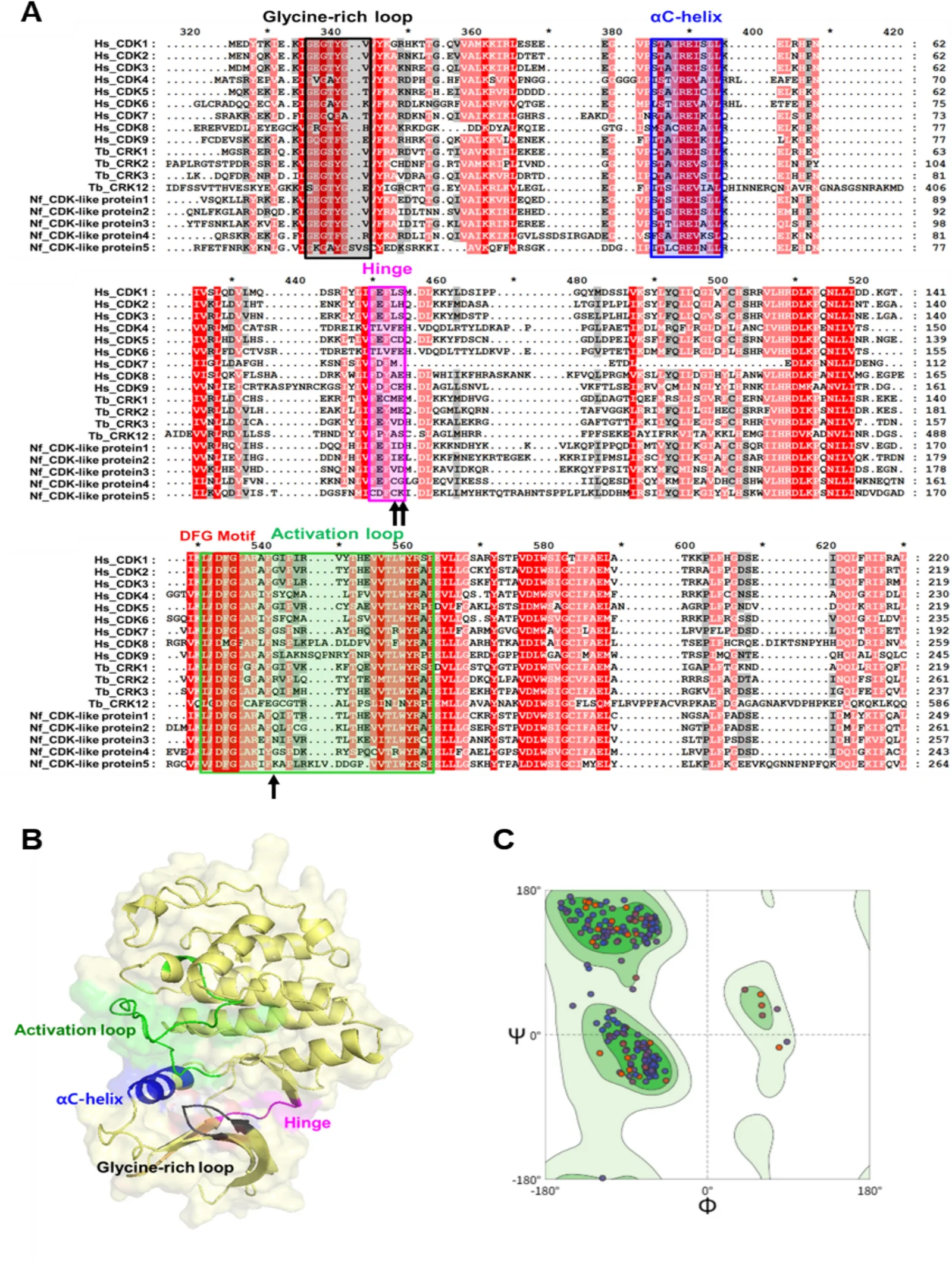
Sequence alignment and structural modeling of Nf_CDK-like protein1. **A** Amino acid sequences of the kinase domains of human CDKs (Hs_CDK1–9), *T. brucei* CRKs (Tb_CRK1, Tb_CRK2, Tb_CRK3, Tb_CRK4, and Tb_CRK6), and *N. fowleri* CDK-like proteins 1–5 were aligned using ClustalX v1.8. Canonical kinase-domain elements are indicated as follows: glycine-rich loop (black), αC-helix (blue), hinge region (pink), and activation loop (green). Black arrows indicate the alignment columns corresponding to Ile110 and Asp111 in the hinge region and Gln183 in the activation loop of Nf_CDK-like protein 1. **B** Three-dimensional homology model of Nf_CDK-like protein 1 generated using SWISS–MODEL. **C** Ramachandran plot of the Nf_CDK-like protein 1 model

Sequence homology analysis utilizing BLASTp identified five distinct CDK-like proteins within the *N. fowleri* proteome. A distinct subgroup comprising Nf_CDK-like protein1 (FDP41_013684), Nf_CDK-like protein2 (FDP41_0117851), and Nf_CDK-like protein3 (FDP41_0002611) exhibited the highest homology to human CDK1/CDK2, sharing approximately 58–61% sequence identity (Fig. 3). Although these three candidates showed comparable levels of homology to human CDKs, Nf_CDK-like protein1 showed the highest similarity to *T. brucei* CRK3 among the three candidates. In the comparative alignment, sequence identities to *T. brucei* CRK3 were 53.9% for Nf_CDK-like protein1, 53.1% for Nf_CDK-like protein2, and 47.6% for Nf_CDK-like protein3. Nf_CDK-like protein1 was, therefore, selected as the representative CDK-like candidate for subsequent structural analyses, based on its relationship to both human CDK1/CDK2 and the protozoan CDK-related kinase CRK3. Multiple sequence alignment mapped the major kinase motifs of Nf_CDK-like protein1, including the glycine-rich loop (Gly38–Val44), αC-helix (Ser73–Leu82), hinge region (Phe107–Asp111), DFG motif (Asp175–Gly177), and activation loop (Leu173–Pro201) (Fig. 4A). In the hinge-proximal region, residues corresponding to Leu83 and Ser84 in human CDK1/2 aligned to Ile110 and Asp111 in Nf_CDK-like protein1, whereas the corresponding positions in *T. brucei* CRK3 were Val102 and Asp103. At the aligned activation-loop position corresponding to Gly154 in human CDK1 and Gly153 in human CDK2, Nf_CDK-like protein1 contained Gln183, whereas *T. brucei* CRK3 contained Gln171. These corresponding residues are indicated by black arrows in the alignment (Fig. 4A). A homology model generated using SWISS–MODEL showed a canonical bilobal kinase fold, with the mapped motifs positioned around the central ATP-binding cleft (Fig. 4B). Ramachandran plot analysis further indicated that 96.9% of residues were located in favored regions (Fig. 4C).

### Docking of prioritized CDK inhibitors to the predicted binding site

**Fig. 5:**
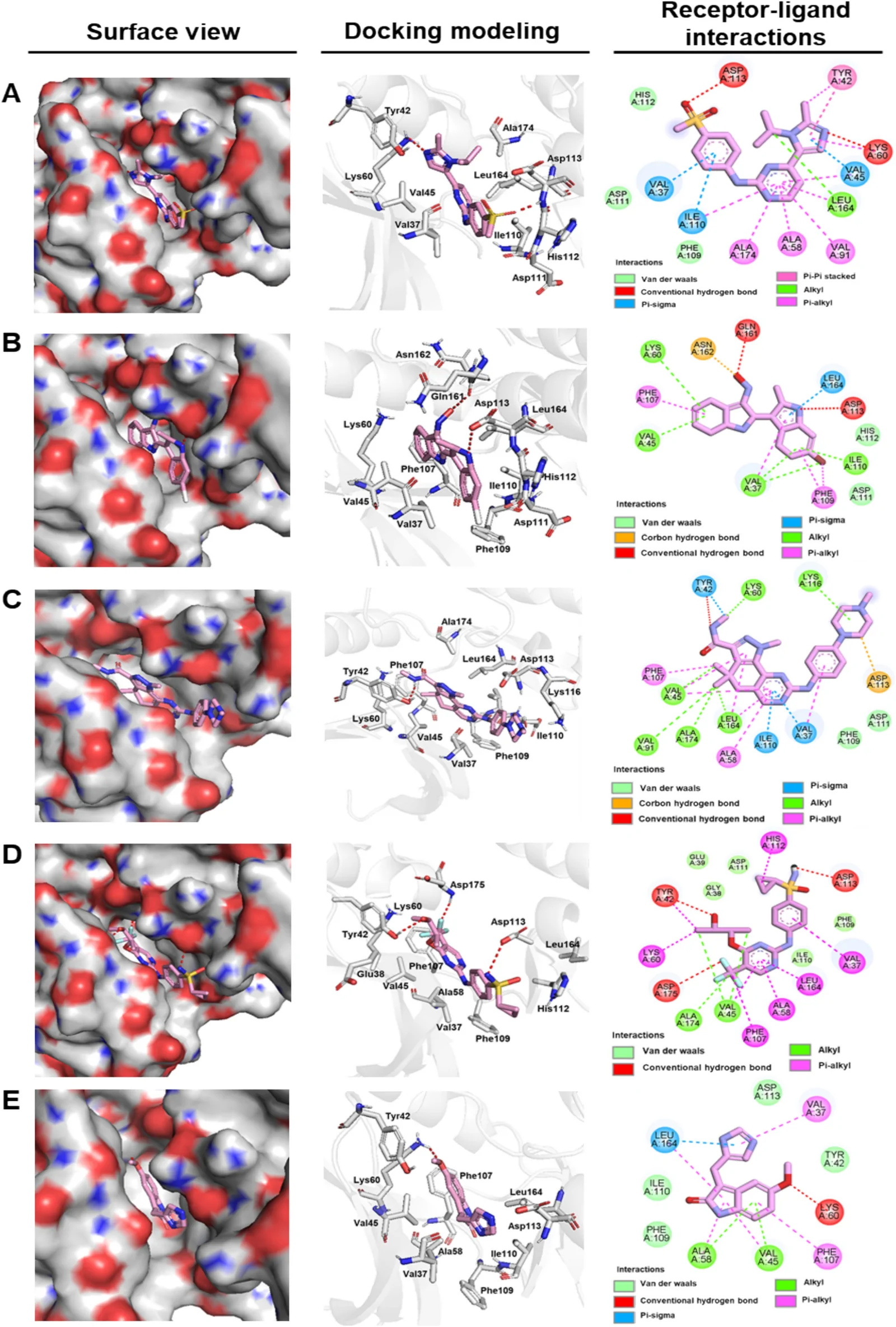
Molecular docking and binding mode analysis of hit compounds in Nf_CDK-like protein1. Predicted binding orientations and molecular interactions of (**A**) AZD5438, (**B**) GSK-3 inhibitor IX, (**C**) Milciclib, (**D**) Roniciclib, and (**E**) SU9516. For each compound, the panels show a surface view of the ATP-binding pocket with the docked ligand (left), a three-dimensional view of protein–ligand interactions (center), and a two-dimensional interaction diagram (right)

Molecular docking using AutoDock Vina placed all five prioritized compounds within the predicted ATP-binding cleft of Nf_CDK-like protein1, and the predicted binding energies and estimated inhibition constants are summarized in Table 3. The docked poses showed a shared interaction pattern centered on Lys60, Tyr42, Asp113, and the hinge-proximal residues Ile110 and Asp111, consistent with occupancy of a common ATP-binding site framework (Fig. 5). Lys60, corresponding to the catalytic Lys33 in human CDK1, formed hydrogen bonds with AZD5438 and SU9516 at distances of 2.80 Å and 2.45 Å, respectively, whereas alkyl contacts were observed with GSK-3 inhibitor IX, milciclib, and roniciclib. In the hinge-proximal region, Asp113 formed hydrogen bonds with AZD5438, roniciclib, and GSK-3 inhibitor IX at distances ranging from 2.06 to 3.40 Å. Tyr42 formed hydrogen bonds with roniciclib and milciclib at 2.63 Å and 3.61 Å, respectively, and showed a π–π interaction with AZD5438. The hinge residues Ile110 and Asp111 were involved in ligand contacts across all five compounds through van der Waals, π–σ, and/or alkyl interactions. Additional recurrent hydrophobic contacts were observed around the pocket periphery. Val37, Val45, and Leu164 interacted with all five compounds, whereas Phe107 and Phe109 contacted four compounds, namely, GSK-3 inhibitor IX, milciclib, roniciclib, and SU9516. Compound-specific interactions included a hydrogen bond between roniciclib and Asp175 in the activation loop at 2.42 Å, and an additional hydrogen bond between GSK-3 inhibitor IX and Gln161 at 3.20 Å. The docking protocol was further evaluated by redocking of reference crystal complexes (PDB IDs: 6GUH, 2WIH, 5IEV, and 3PYO), which yielded ligand RMSD values of 1.00–1.61 Å and pocket RMSD values of 1.38–1.98 Å (Additional file 2: Figure S2).

**Table 3:** Predicted binding affinities and estimated inhibition constants (K_i_) of the five prioritized hit compounds docked to Nf_CDK-like protein1

No	Compounds	Binding affinity (Kcal/mol)	Inhibition constant (K_i_) µM
1	AZD5438	− 8.9	0.299
2	GSK 3 inhibitor Ⅸ	− 10.0	0.046
3	Milciclib	− 10.2	0.033
4	Ronciclib	− 9.1	0.213
5	SU9516	− 7.7	2.27

### Molecular dynamics analysis of protein–ligand complex stability

**Fig. 6:**
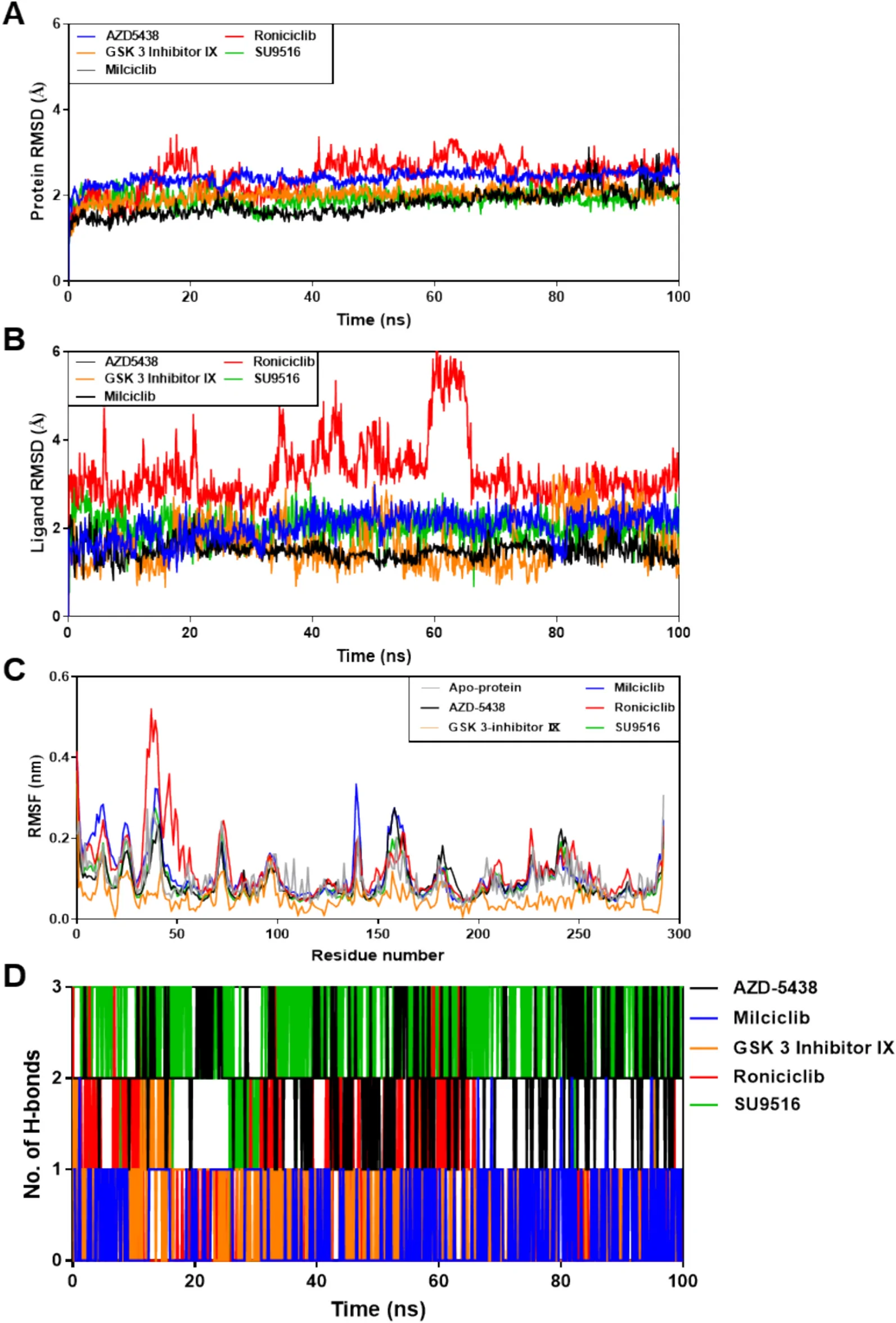
Stability assessment of Nf_CDK-like protein 1–ligand complexes by 100 ns molecular dynamics simulations. **A** Backbone RMSD trajectories. **B** Ligand RMSD values calculated after least-squares fitting to the protein backbone. **C** Per-residue Cα RMSF profiles. **D** Protein–ligand hydrogen-bond counts during the simulations

To evaluate binding stability and protein dynamics, 100 ns molecular dynamics simulations were performed for each Nf_CDK-like protein1–ligand complex, and the trajectories were analyzed in terms of protein backbone RMSD, protein-aligned ligand RMSD, per-residue Cα RMSF, and protein–ligand hydrogen bonds (Fig. 6A–D). Following the initial equilibration period, backbone RMSD values for all complexes remained within a relatively narrow range, with an overall fluctuation of approximately 1.77 Å, indicating that the protein structures were maintained within a stable conformational window under the simulation conditions (Fig. 6A). Ligand pose stability was further assessed using ligand RMSD after alignment to the protein backbone (Fig. 6B). The AZD5438-, GSK-3 inhibitor IX-, milciclib-, and SU9516-bound complexes stabilized at mean ligand RMSD values of approximately 2.03 Å, 1.66 Å, 1.49 Å, and 2.02 Å, respectively, whereas the roniciclib-bound complex showed a higher mean ligand RMSD of 3.22 Å, with maxima reaching 5.77 Å. The Cα RMSF profiles were broadly similar across the five complexes and the apo protein, with higher fluctuations primarily localized to surface-exposed loop regions (Fig. 6C). In particular, residues G38–V44 and D175–E202 showed increased RMSF values in the milciclib-, roniciclib-, and SU9516-bound systems. Protein–ligand hydrogen bonding was quantified as the time-dependent number of hydrogen bonds throughout the simulations (Fig. 6D). The mean numbers of hydrogen bonds were 2.1 for AZD5438, 0.4 for GSK-3 inhibitor IX, 0.3 for milciclib, 0.6 for roniciclib, and 2.6 for SU9516. Under the present simulation conditions, AZD5438 and SU9516 maintained higher mean hydrogen-bond counts than the other three ligands.

## Discussion

Primary amoebic meningoencephalitis (PAM) caused by *Naegleria fowleri* is a rapidly progressive and highly fatal infection for which effective treatment options remain limited [1–7]. In this study, screening of a CDK inhibitor library identified several compounds with submicromolar activity against *N. fowleri*, and subsequent sequence- and structure-based analyses supported Nf_CDK-like protein1 as a plausible CDK-like kinase target candidate.

The five prioritized compounds, AZD5438, GSK-3 inhibitor IX, milciclib, roniciclib, and SU9516, showed IC_50_ values of 0.30–0.88 µM against *N. fowleri* trophozoites (Table 2). These findings suggest that CDK-related pathways may be relevant to anti-amoebic drug discovery in *N. fowleri*. The limited selectivity in J774A.1 cells indicates that these compounds remain chemical probes rather than optimized therapeutics (Additional file 1: Table S2).

CDK/CRK family kinases are important regulators of cell-cycle progression and proliferation in other protozoan parasites [21–25]. In *Trypanosoma brucei*, CRK1 and CRK3 have been associated with G1/S- and G2/M-related regulation, respectively, and CRK1 has also been shown to regulate intracellular trafficking through Sec31 phosphorylation [21, 22, 36]. CRK2 has additionally been implicated in posterior cytoskeletal morphogenesis and S-phase-related regulation, whereas CRK4 and CRK6 appear to play more auxiliary roles in supporting cell-cycle progression [37, 38]. In *Leishmania mexicana*, CRK3 has been described as an essential cdc2-related kinase, and several CDK inhibitor classes showed both CRK3 inhibitory activity and anti-leishmanial effects [23]. Leishmanial GSK-3 has likewise been investigated as a kinase target [25]. Taken together, these previous studies indicate that proliferation-associated kinases are functionally important in protozoan parasites and can be approached pharmacologically [21–25].

Among the five identified *N. fowleri* CDK-like proteins, Nf_CDK-like protein1 showed 61.7% and 60.1% sequence identity to human CDK2 and CDK1, respectively, and 53.9% sequence identity to *T. brucei* CRK3 in the comparative analysis (Fig. 3). In *N. fowleri*, kinase-related studies have mainly focused on pathogenicity-associated processes [39, 40], whereas studies directly addressing CDK-like kinases remain limited. The present study, therefore, suggests a possible CDK-like regulatory axis in *N. fowleri*. In addition, sequence and docking analyses showed that Nf_CDK-like protein1 retained a CDK-like ATP-binding architecture but had a local hinge-region feature distinct from human CDK1/2. In the hinge-proximal region, Nf_CDK-like protein1 contained Ile110 and Asp111, and *T. brucei* CRK3 contained Val102 and Asp103, whereas the corresponding positions in human CDK1/2 were Leu83 and Ser84 (Fig. 4A). This pattern indicates that Nf_CDK-like protein1 shares a local hinge-region feature with *T. brucei* CRK3 that is distinct from human CDK1/2. Whether this feature is conserved across protozoan parasites or restricted to selected lineages will require broader comparative analysis. Recent studies in *N. fowleri* have employed homology modeling, molecular docking, and molecular dynamics simulations to assess candidate targets and ligand-binding stability, supporting the structure-based workflow used in the present study [41, 42]. MD simulations complemented the docking analysis by showing overall dynamic stability of the protein–ligand complexes, together with ligand-dependent differences in pose behavior and hydrogen-bonding patterns over time. These results suggest that Nf_CDK-like protein1 can accommodate multiple CDK inhibitor scaffolds within its binding pocket. Additional biochemical target-validation studies using purified protein will be required to determine whether Nf_CDK-like protein1 is directly inhibited by these compounds and whether it represents a primary intracellular target. Further studies on cyclins and their potential pairing partners will also be needed to define the biological context of this putative pathway.

This study identified five CDK inhibitor scaffolds with submicromolar activity against *N. fowleri* and suggested Nf_CDK-like protein1 as a CDK-like kinase target candidate. These results provide useful information for further studies on CDK-like pathways in *N. fowleri* and for the development of selective inhibitors.

## Conclusions

In this study, we identified several CDK inhibitor scaffolds with submicromolar activity against *N. fowleri* and proposed Nf_CDK-like protein1 as a plausible target candidate through integrated phenotypic screening and structure-based analyses. Although further validation and compound optimization will be required, the present findings support CDK-like pathways as a potentially relevant molecular axis in *N. fowleri*. The prioritized inhibitors may, therefore, serve not only as starting points for future parasite-biased inhibitor development but also as tool compounds for investigating CDK-related biology in this pathogen.

## Supplementary Information

Additional file1 (DOCX 19 KB)

Additional file2 (DOCX 1167 KB)

## Data Availability

Data supporting the main conclusions of this study are included in the manuscript.

## Abbreviations

ADT: AutoDockTools
ATP: Adenosine triphosphate
BLASTp: Protein–protein Basic Local Alignment Search Tool
CC_50_: Half-maximal cytotoxic concentration
CDK: Cyclin-dependent kinase
CNS: Central nervous system
CRK: Cdc2-related kinase
DMSO: Dimethyl sulfoxide
FBS: Fetal bovine serum
IC_50_: Half-maximal inhibitory concentration
MD: Molecular dynamics
PAM: Primary amebic meningoencephalitis
PDB: Protein Data Bank
PDBQT: Protein Data Bank, Partial Charge, and Atom Type
PME: Particle mesh Ewald
RMSD: Root mean square deviation
RMSF: Root mean square fluctuation
SI: Selectivity index
