# Supplementary material for: Cyclin-dependent kinase-inhibitor screening and structural analysis identify a CDK-like kinase candidate in Naegleria fowleri
Source: Parasit Vectors. 2026 Jun 24;19:386. doi: 10.1186/s13071-026-07525-8 (PMC13556005; doi:10.1186/s13071-026-07525-8)
Supplement: Supplementary file 1 [file 13071_2026_7525_MOESM1_ESM.docx]

**Table S1.** IC₅₀ values of the remaining 18 active hit compounds against *N. fowleri*.

| **No.** | **Compounds** | **IC_50_ (µM) ± SD** |
| --- | --- | --- |
| 1 | SEL120-34A (monohydrochloride) | 3.125 ± 0.898 |
| 2 | Dinaciclib | 9.67 ± 1.23 |
| 3 | Abemaciclib (methanesulfonate) | 2.834 ± 0.45 |
| 4 | AZD4573 | 3.12 ± 1.52 |
| 5 | Flavopiridol | 6.25 ± 0.56 |
| 6 | Simurosertib | 2.594 ± 0.2 |
| 7 | (E/Z)-Zotiraciclib | 0.378 ± 0.63 |
| 8 | CGP60474 | 0.0488 ± 0.02 |
| 9 | BMS-265246 | 3.125 ± 0.2 |
| 10 | Trilaciclib (hydrochloride) | 0.5411 ± 0.033 |
| 11 | FN-1501 | 4.918 ± 0.52 |
| **12** | ON123300 | 1.142 ± 1.03 |
| 13 | CDK5 inhibitor 20-223 | 6.847 ± 0.18 |
| 14 | CDKI-73 | 0.9732 ± 0.52 |
| 15 | TL12-186 | 1.958 ± 0.68 |
| 16 | CDK9-IN-7 | 3.633 ± 0.98 |
| 17 | Casein Kinase inhibitor A51 | 1.5625 ± 0.23 |
| 18 | NU6027 | 7.081 ± 0.69 |

Data are presented as mean ± SD from independent duplicate experiments.

**Table S2.** Cytotoxicity of prioritized hit compounds and reference drugs in J774A.1 cells. CC₅₀ values and selectivity indices (SI = CC₅₀/IC₅₀) are shown.

| **No.** | **Compound** | **CC_50_ (µM) ± SD** | **SI** |
| --- | --- | --- | --- |
| 1 | AmphotericinB | ≥ 200 | 2247.191 |
| 2 | Miltefosine | 92.5 ± 13.43 | 2.69 |
| 3 | AZD-5438 | 1.292 ± 0.095 | 2.78 |
| 4 | GSK 3 inhibitor Ⅸ | 2.234 ± 0.243 | 2.85 |
| 5 | Milciclib | 0.8218 ± 0.35 | 2.71 |
| 6 | Ronciclib | 1.265 ± 0.106 | 1.43 |
| 7 | SU9516 | 0.8691 ± 0.4861 | 1.03 |

Data are presented as mean ± SD from independent duplicate experiments.
