# Supplementary material for: Cyclin-dependent kinase-inhibitor screening and structural analysis identify a CDK-like kinase candidate in Naegleria fowleri
Source: Parasit Vectors. 2026 Jun 24;19:386. doi: 10.1186/s13071-026-07525-8 (PMC13556005; doi:10.1186/s13071-026-07525-8)
Supplement: Supplementary file 2 [file 13071_2026_7525_MOESM2_ESM.docx]

**Fig. S1**. **Cytotoxicity profiles of the prioritized hit compounds and reference drugs in murine macrophage J774A.1 cells.** Cell viability curves for (A) amphotericin B, (B) miltefosine, (C) AZD5438, (D) GSK-3 inhibitor IX, (E) milciclib, (F) roniciclib, and (G) SU9516 after 48 h of treatment.

**Fig. S2. Validation of the molecular docking protocol by redocking analysis using reference crystal complexes.** Superposition of the top-ranked docked poses and the corresponding crystallographic ligand conformations for (A) AZD5438 (PDB ID: 6GUH), (B) milciclib (PDB ID: 2WIH), (C) roniciclib (PDB ID: 5IEV), and (D) SU9516 (PDB ID: 3PYO). In each panel, the docked ligand is shown in grey and the crystallographic ligand is shown in green. Ligand RMSD and pocket RMSD values are indicated for each complex.
